# Eat1-Like Alcohol Acyl Transferases From Yeasts Have High Alcoholysis and Thiolysis Activity

**DOI:** 10.3389/fmicb.2020.579844

**Published:** 2020-10-29

**Authors:** Constantinos Patinios, Lucrezia Lanza, Inge Corino, Maurice C. R. Franssen, John Van der Oost, Ruud A. Weusthuis, Servé W. M. Kengen

**Affiliations:** ^1^Laboratory of Microbiology, Department of Agrotechnology and Food Sciences, Wageningen University and Research, Wageningen, Netherlands; ^2^Laboratory of Bioprocess Engineering, Department of Agrotechnology and Food Sciences, Wageningen University and Research, Wageningen, Netherlands; ^3^Laboratory of Organic Chemistry, Department of Agrotechnology and Food Sciences, Wageningen University and Research, Wageningen, Netherlands

**Keywords:** alcoholysis, thiolysis, α/β-hydrolase, alcohol acyl transferase (AAT), yeast, ester, *Clostridium beijerinckii*

## Abstract

Esters are important flavor and fragrance compounds that are present in many food and beverage products. Many of these esters are produced by yeasts and bacteria during fermentation. While ester production in yeasts through the alcohol acyl transferase reaction has been thoroughly investigated, ester production through alcoholysis has been completely neglected. Here, we further analyze the catalytic capacity of the yeast Eat1 enzyme and demonstrate that it also has alcoholysis and thiolysis activities. Eat1 can perform alcoholysis in an aqueous environment *in vitro*, accepting a wide range of alcohols (C2–C10) but only a small range of acyl donors (C2–C4). We show that alcoholysis occurs *in vivo* in several Crabtree negative yeast species but also in engineered *Saccharomyces cerevisiae* strains that overexpress Eat1 homologs. The alcoholysis activity of Eat1 was also used to upgrade ethyl esters to butyl esters *in vivo* by overexpressing Eat1 in *Clostridium beijerinckii*. Approximately 17 mM of butyl acetate and 0.3 mM of butyl butyrate could be produced following our approach. Remarkably, the *in vitro* alcoholysis activity is 445 times higher than the previously described alcohol acyl transferase activity. Thus, alcoholysis is likely to affect the ester generation, both quantitatively and qualitatively, in food and beverage production processes. Moreover, mastering the alcoholysis activity of Eat1 may give rise to the production of novel food and beverage products.

## Introduction

The α/β-hydrolase fold superfamily of enzymes belongs to one of the largest groups of structurally related enzymes sharing a typical α/β-sheet with a conserved active site. Members of this family have diverse catalytic functions, including peptidase (EC 3.4), alcohol acyl transferase (AAT) (EC 2.3.1), esterase (EC 3.1.1), thioesterase (EC 3.1.2), lipase (EC 3.1.1.3), and others (Nardini and Dijkstra, 1999; Holmquist, 2000). In fact, members of this superfamily can catalyze 17 different reactions through the same Ser-His-Asp catalytic triad (Rauwerdink and Kazlauskas, 2015). Because of their wide portfolio of catalytic reactions, their high catalytic activity, their high stability and their high regio- and stereoselectivity, some α/β-hydrolase fold enzymes are widely used in biotechnology (Bornscheuer and Kazlauskas, 2006). Applications range from the production of flavors and fragrances such as butyl butyrate and cinnamyl propionate in the food industry, the production of biologically potent pharmaceutical chemicals, such as (R)-indanol and lutein dipalmitate, and the synthesis or degradation of polymers such as poly (β-caprolactone) or polyhydroxyalkanoates, respectively (Priyanka et al., 2019).

**FIGURE 1:**
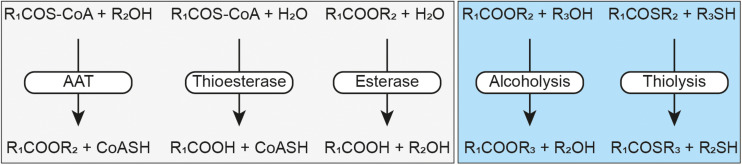
Schematic representation of the Eat1 activities. Alcohol acyl transferase (AAT), thioesterase and esterase activities have previously been described by Kruis et al. (2017) (light gray box). Alcoholysis and thiolysis are described by this study (light blue box). An acyl-CoA is represented by RCOS-CoA, a free CoA by CoASH, an ester by RCOOR, a thioester by RCOSR, an alcohol by ROH and a thiol by RSH.

A new member of the α/β-hydrolase fold superfamily is the Eat1 protein family. Eat1 was recently discovered in ester-producing yeast species, such as *Kluyveromyces marxianus*, *Kluyveromyces lactis*, and *Wickerhamomyces anomalus*, and was identified as the main enzyme responsible for ethyl acetate formation in these yeasts (Kruis et al., 2017; Löbs et al., 2018). Ethyl acetate synthesis by Eat1 is catalyzed through the transfer of the acetate moiety of acetyl-CoA to a free molecule of ethanol in an AAT reaction (Figure 1). It is hypothesized that the role of Eat1 is to regenerate the free CoA pool in the cell during iron or oxygen limitation through ethyl acetate production (Fredlund et al., 2004; Urit et al., 2012; Löser et al., 2014; Löser et al., 2015; Kruis et al., 2018). This hypothesis was strengthened by the localization of Eat1 in the mitochondria of *K. lactis* and *K. marxianus* and its upregulated expression in *W. anomalus* during iron-limited conditions (Kruis et al., 2017, 2018; Löbs et al., 2018).

**FIGURE 2:**
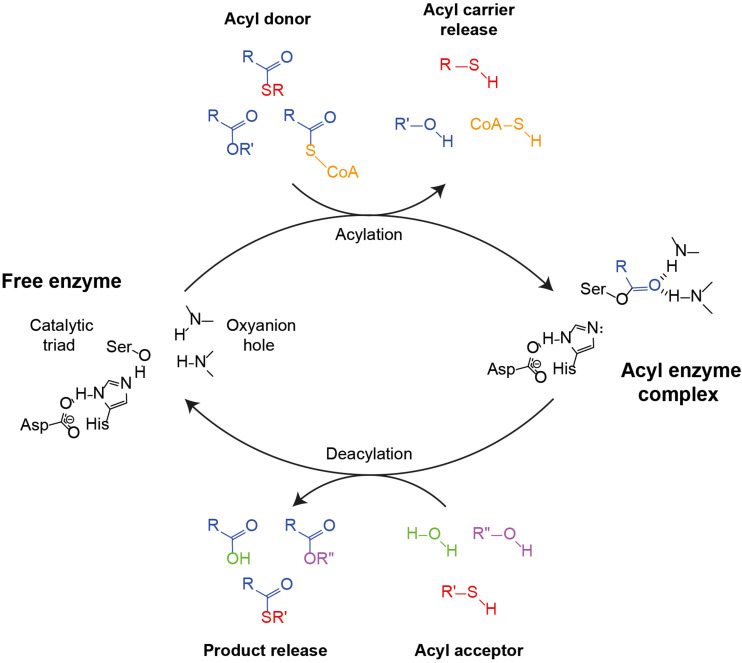
Canonical esterase mechanism demonstrating AAT, hydrolysis, alcoholysis or thiolysis reactions. The catalytic triad (Asp–His–Ser) along with the oxyanion hole (two amide N-Hs) are indicated in the free enzyme state (left). The first step of the cycle is the binding of the acyl moiety of an acyl-CoA, an ester or a thioester (colored yellow, blue, or red, respectively), followed by acylation and the release of the acyl carrier and the formation of the acyl enzyme complex (right). Next, depending on whether an alcohol or a thiol is present in the aqueous medium, hydrolysis, ester formation or thioester formation may occur. In the case of esterase or thioesterase, water (green) binds to the acyl enzyme complex, causing the release of the acid and the restoration of the free enzyme state. In the case of AAT or alcoholysis, an alcohol (magenta) binds to the acyl enzyme complex, which causes the release of an ester. The difference between AAT and alcoholysis is defined by the acyl carrier molecule as a CoA or an alcohol, respectively. Likewise, if a thiol (red) nucleophile is present, the product of the cycle will be a thioester.

In addition to its AAT activity, Eat1 can also catalyze esterase and thioesterase activities (Figure 1), both through hydrolysis, in an aqueous environment (Kruis et al., 2017). This is not surprising as all three activities follow the canonical esterase mechanism for hydrolysis (Figure 2). Remarkably, however, Eat1 was shown to favor ethanol over water as a nucleophile, resulting in a preference for the AAT activity over the competing hydrolysis reactions at elevated alcohol concentrations (Kruis et al., 2017). This feature is unique among the α/β-hydrolase fold enzymes as only a small fraction of these enzymes favors alcohols over water in an aqueous environment (Rauwerdink and Kazlauskas, 2015). Therefore, based on the observation that Eat1 can accept both esters and thioesters as acyl donors, along with its preference for ethanol over water as acyl acceptor, we hypothesized that Eat1 might also be able to catalyze alcoholysis and thiolysis (Figure 1).

Alcoholysis is a transesterase reaction in which the alcohol moiety of an ester is replaced by another alcohol following the general equation: R_1_COOR_2_ + R_3_OH → R_1_COOR_3_ + R_2_OH (Figure 1). Alcoholysis occurs both in aqueous and in non-aqueous environments using lipases or esterases and has been widely used by the industry for biodiesel production (Lotti et al., 2018). Likewise, thiolysis (or transthioesterification) describes the production of a thioester by donating an acyl group from an acyl donor to a thiol acceptor. The acyl donor can be either an ester or a thioester, but the former is generally used: R_1_COOR_2_ + R_3_SH → R_1_COSR_3_ + R_2_OH (Zaks and Klibanov, 1985; Cavaille-Lefebvre and Combes, 1997).

While ester production in yeasts has been thoroughly investigated through the AAT reaction, ester production through alcoholysis has been completely neglected. Alcoholysis, can play an important role in the production and distribution of esters in yeasts and consequently affect the final product quality of yeast-derived food and beverage products like beer and wine. Here we investigated the catalytic capacity of the Eat1 α/β-hydrolase with respect to alcoholysis and (to a minor extent) thiolysis. We could show that the Eat1 enzyme exhibits a high alcoholysis activity using a broad range of alcohols. In addition, we show that alcoholysis can also play a role *in vivo* in several yeast species and that expression of Eat1 leads to alcoholysis in engineered yeast species and in an engineered bacterium (*Clostridium beijerinckii*). The discovery of the high alcoholysis side activity of Eat1 sheds new light on the production and variety of short chain esters in food and beverage products and might open new research lines for the industrial production of sustainable short chain esters.

## Materials and Methods

### Microbial Strains and Cultivation Conditions

The strains used in this study are given in Table 1. Transformed *Escherichia coli* was grown at 37°C on LB agar plates containing 50 mg L^–1^ kanamycin or spectinomycin or cultured in liquid LB media containing 100 mg L^–1^ kanamycin or spectinomycin. For plasmid construction and propagation, chemically competent *E. coli* NEB^®^ 5-alpha was used and for protein expression, BL21 (DE3) competent *E. coli* was used following the manual provided by the manufacturer (NEB).

**TABLE 1:** Strains used in this study.

Strain	Genotype	Source
*Escherichia coli* NEB 5-alpha	*fhuA2*Δ*(argF-lacZ)U169 phoA glnV44*Φ*80*Δ*(lacZ)M15 gyrA96 recA1 relA1 endA1 thi-1 hsdR17*	New England Biolabs (NEB)
*Escherichia coli* BL21 (DE3)	*fhuA2 [lon] ompT gal (*λ *DE3) [dcm]*Δ*hsdS*λ *DE3* = λ *sBamHIo*Δ*Eco*RI*-B int:(lacI:PlacUV5:T7 gene1) i21*Δ*nin5*	NEB
*Saccharomyces cerevisiae* CEN.PK2-1D	*MATalpha, his3D1, leu2-3_112, ura3-52, trp1-289, MAL2-8c, SUC2*	Entian and Kötter (2007)
*Wickerhamomyces anomalus* DSM 6766	Wild type	Leibniz-Institut DSMZ
*Wickerhamomyces ciferrii* CBS 111	Wild type	Centraalbureau voor Schimmelcultures (CBS)
*Kluyveromyces marxianus* DSM 5422	Wild type	Leibniz-Institut DSMZ
*Kluyveromyces lactis* CBS 2359	Wild type	CBS
*Cyberlindnera jadinii* DSM 2361	Wild type	Leibniz-Institut DSMZ
*Cyberlindnera fabianii* CBS 5640	Wild type	CBS
*Hanseniaspora uvarum* CECT 11105	Wild type	Colección Española de Cultivos Tipo (CECT)
*Clostridium beijerinckii* NCIMB 8052	Wild type	Scotcher and Bennett (2005)

The wild type (WT) yeast strains were grown in 50-ml Falcon tubes containing 10 ml of either YPD medium (10 g L^–1^ yeast extract, 20 g L^–1^ peptone, and 20 g L^–1^ glucose), yeast minimal medium (YMM) or YMM without iron supplementation (YMM-no-iron) as described previously (Kruis et al., 2018). *In vivo* alcoholysis was enabled by adding 5 mM butyl butyrate to YMM-no-iron. As a control for esterification, WT strains were also grown in YMM-no-iron containing 5 mM butyrate. Cultures were grown in triplicate for 48 h at 30°C on a shaking platform at 200 rpm. Samples were taken after 48 h and stored at −20°C until further use.

*Saccharomyces cerevisiae* strains harboring the pCUP1 variants were grown in 50-mL Falcon tubes containing 10 mL YMM medium with the required growth factors to supplement the auxotrophic requirements of the strains (75 mg L^–1^ tryptophan, 500 mg L^–1^ leucine, and 125 mg L^–1^ histidine). Expression of the Eat1 homologs and *in vivo* alcoholysis was assessed by adding 100 μM CuSO_4_ and 5 mM butyl butyrate to the growth medium. Control cultures were grown in medium containing 5 mM butyrate. Cultures were incubated at 30°C for 24 h on a shaking platform at 200 rpm and sampled once at the end of the fermentation. Samples were stored at 20°C until further use.

Transformed *C. beijerinckii* strains were grown anaerobically on mCGM agar medium (5 g L^–1^ yeast extract, 5.51 mM KH_2_PO_4_, 4.31 mM K_2_HPO_4_, 1.62 mM MgSO_4_, 0.036 mM FeSO_4_, 17.11 mM NaCl, 15.14 mM L-asparagine, 15.14 mM (NH_4_)_2_SO_4_, 1.03 mM L-cysteine, and 69.4 mM D(+)-glucose) or fermented in GAPES liquid medium (2.5 g L^–1^ yeast extract, 7.35 mM KH_2_PO_4_, 3.50 mM K_2_HPO_4_, 4.06 mM MgSO_4_, 0.02 mM FeSO_4_, 1.30 mM pABA, 37.65 mM CH_3_CO_2_NH_4_, 331.02 mM D(+)-glucose, and 0.99 mM L-cysteine; Gapes et al., 1996) containing 650 mg L^–1^ spectinomycin. For *in vivo* alcoholysis, 0, 10, 20, 50 or 100 mM of ethyl acetate or ethyl butyrate was added to the growth medium and the cultures were grown for 96 h. Samples were taken at 48, 72, and 96 h and stored at −20°C until further use.

### Plasmid Construction

Table 2 lists all the plasmids used in this study. The pET26b:harmWanomala_5543-His and pCUP1 plasmids were derived from Kruis et al. (2017). To construct pCOSCB3:WanEat1 the WT *W. anomalus* Eat1 sequence was amplified through PCR from the genomic DNA of *W. anomalus* DSM 6766 and introduced downstream of the strong constitutive thiolase promoter derived from *C. beijerinckii* NCIMB 8052. Individual fragments containing the spectinomycin resistance gene, the pAMB1 ori and the ColE1 ori were also amplified through PCR and assembled through NEBuilder^®^ HiFi DNA Assembly. For pCOSCB:EV, the WT *W. anomalus* Eat1 gene was omitted from the plasmid.

**TABLE 2:** Plasmids used in this study.

Plasmid	Host, relevant gene	Source
pCUP1:EV	*S. cerevisiae*, Empty vector	Kruis et al. (2017)
pCUP1:WanEat1	*S. cerevisiae*, *W. anomalus* Eat1	Kruis et al. (2017)
pCUP1:WciEat1	*S. cerevisiae*, *W. ciferii* Eat1	Kruis et al. (2017)
pCUP1:KmaEat1	*S. cerevisiae*, *K. marxianus* Eat1	Kruis et al. (2017)
pCUP1:KlaEat1	*S. cerevisiae*, *K. lactis* Eat1	Kruis et al. (2017)
pCUP1:CjaEat1	*S. cerevisiae*, *C. jadinii* Eat1	Kruis et al. (2017)
pCUP1:CfaEat1	*S. cerevisiae*, *C. fabianii* Eat1	Kruis et al. (2017)
pCUP1:HuvEat1	*S. cerevisiae*, *H. uvarum* Eat1	Kruis et al. (2017)
pCUP1:SceEat1	*S. cerevisiae*, *S. cerevisiae* Eat1	Kruis et al. (2017)
pET26b:EV	*E. coli*, Empty vector	Novagen
pET26b:harmWanomala_ 5543-His	*E. coli*, codon harmonized *W. anomalus* Eat1	Kruis et al. (2017)
pCOSCB:EV	*C. beijerinckii*, Empty vector	This study
pCOSCB3:WanEat1	*C. beijerinckii*, *W. anomalus* Eat1	This study

### Chemicals and Reagents

Unless otherwise specified, all the chemical reagents were purchased from Sigma-Aldrich. 1-Octen-3-ol, 1-phenylethanol, 2-phenylethanol, *cis*-3-hexenol, citronellol, geraniol, linalool, *trans*-2-hexenol, and their acetate esters were kindly provided by Axxence Aromatic GmbH, Germany.

### WanEat1 Expression and Purification

To purify the WanEat1 protein, a previously established protocol was used (Mohanraju et al., 2018). Briefly, *E. coli* BL21 (DE3) bearing pET26b:harmWanomala_5543-His was grown in 1 L LB medium containing 100 mg L^–1^ kanamycin at 37°C and shaken at 120 rpm. When an OD_600_ of 0.5–0.6 was reached, the cultures were chilled on ice water for 15 min before they were induced with 0.2 mM IPTG. The cultures were then incubated overnight (∼16 h) at 20°C and shaking at 120 rpm. After overnight incubation, the cells were harvested by centrifuging the culture for 15 min at 6,000 × *g* at 4°C. The cell pellet was washed with 50 ml of 50 mM potassium phosphate buffer (KPi, pH 7.5), centrifuged for 15 min at 6,000 × *g* at 4°C and the cell pellet was stored at −20°C until use.

For cell lysis, the cell pellet was resuspended in 20 mL of HA buffer (50 mM KPi, 300 mM NaCl, pH 8.0, and 20 mM imidazole) containing 1 mini tablet cOmplete^TM^ protease inhibitor for every 10 mL of HA buffer. Cells were disrupted by sonication using a VS 70 T tip (Bandelin SONOPLUS HD) using the following setup: 25% amplitude, 10 min total time and 1 s ON/2 s OFF. The cell lysate was centrifuged at 4°C at 30,000 × *g* for 45 min. The supernatant was collected and filtered through a 0.22 μm membrane filter and then used for protein purification.

The cell-free extract was subjected to Q-sepharose Fast Performance Liquid (FPLC) purification through ÄKTA go Protein Purification System (GE Healthcare Life Sciences). The first purification step involved loading the cell-free extract on a 1-mL His Trap^TM^ HP column (GE Healthcare Life Sciences) equilibrated with HA buffer and then eluted by washing the column with HB buffer (50 mM KPi, 300 mM NaCl, 500 mM imidazole, pH 8.0). The fractions containing the protein were collected, combined and diluted five times with CA buffer (50 mM KPi, pH 7.0). The diluted protein was loaded on a 1-mL HiTrap SP HP column (GE Healthcare Life Sciences) equilibrated with CA buffer and eluted by a NaCl gradient in CA buffer (50 mM KPi, pH 7.0, 1 M NaCl). The fractions containing the highest protein content were combined and used for further analysis.

SDS-PAGE was used to analyze the purity of the protein samples. The protein samples were denatured by heating at 98°C for 5 min in 4× Laemmli Sample Buffer (Bio-Rad) and centrifuged at 10,000 rpm for 30 s. Proteins were then separated on Mini-PROTEAN^®^ TGX^TM^ precast gel (Bio-Rad) at 20 mA for 45 min and stained using Page Blue^TM^ protein staining solution (Thermo Fisher Scientific).

### Enzyme Assays

All enzyme assays were performed at 30°C in a phosphate buffer (50 mM KPi, 150 mM NaCl, pH 7.5).

The 4-nitrophenol release assay was performed by measuring the release of 4-nitrophenol from 0.1 mM 4-nitrophenyl acetate (diluted from a 200 mM stock in DMSO) at 405 nm using a temperature-controlled U-2010 spectrophotometer (Hitachi). The initial slope was determined as the absorption per min of 4-nitrophenol at 405 nm and the amount of 4-nitrophenol was calculated from a calibration curve of 4-nitrophenol (*r*^2^ ≥ 0.99) at 30°C in a phosphate buffer (50 mM KPi, 150 mM NaCl, pH 7.5).

To assess alcoholysis and thiolysis, the 4-nitrophenol release assay was performed in the presence of different concentrations of ethanol, 1-butanol, 1-hexanol, ethanethiol or butanethiol (0–80 mM). The reaction was initiated by adding purified WanEat1 protein (0.69 μg ml^–1^ final concentration).

To analyze the reaction mixture for ester (alcoholysis) or thioester (thiolysis) formation during the 4-nitrophenol release assay, we repeated the assay as described above but we used 2.5 mM 4-nitrophenyl acetate and 2.5 mM of the alcohols or thiols. Every 10 s and for a total of 3 min, 100 μL of the reaction mixture was recovered and mixed with 100 μL stop solution (0.1 N H_2_SO_4_ with 10 mM acetone as internal standard) and 100 μL n-hexane, vortexed vigorously and let the extraction to proceed for 15 min. The hexane layer was then analyzed by GC.

To assess whether the inhibition of Eat1 by alcohols or thiols was reversible, 11.04 μg mL^–1^ purified WanEat1 protein was first briefly incubated with 80 mM ethanol, 1-butanol, 1-hexanol, ethanethiol or butanethiol before diluting to 5 mM final alcohol or thiol concentration. The final enzyme concentration was the same (0.69 μg mL^–1^) as in the standard assay. The reaction was initiated by adding 0.1 mM 4-nitrophenyl acetate. The final reaction volume for the 4-nitrophenol release and inhibition assays was 1 mL.

*In vitro* alcoholysis and thiolysis assays were performed in triplicate in gas tight glass vials at 30°C in the presence of 13.98 μg mL^–1^ purified protein. The total volume of the reaction was 250 μL and was performed in a phosphate buffer (50 mM KPi, 150 mM NaCl, pH 7.5) supplemented with 2.5 mM of the ester and 2.5 mM of the alcohol or thiol. The reaction mixture was incubated at 30°C for 10 min. Then, the reaction was initiated by adding the enzyme to the reaction mixture and the reaction was terminated after 5 min of incubation by adding 250 μL stop solution (0.1 N H_2_SO_4_). A total of 10 mM acetone was included in the stop solution as internal standard. Extraction of the mixture of substrates and products was done by adding 250 μL n-hexane. The sample vial was vortexed vigorously and the extraction proceeded for 15 min. Following, the n-hexane layer was used for GC analysis.

### Analytical

For *in vitro* alcoholysis and thiolysis, esters, thioesters, alcohols or thiols recovered by n-hexane extraction were analyzed on a Shimadzu GC 2010 gas chromatograph equipped with an AOC 20i + s autosampler (Shimadzu). A total of 1 μL of the sample was injected on a DB-WAX UI column (30 m length, 0.53 mm inner diameter, 1 μm film thickness, Agilent) with a split ratio 1:20. The column temperature was kept at 70°C for 1 min followed by an increase to 125°C at a rate of 50°C min^–1^ and then followed by an increase to 230°C at a rate of 50°C min^–1^ where the temperature was kept at 230°C for 1 min.

*In vivo* alcoholysis by WT yeasts or by transformed *S. cerevisiae* was analyzed by taking a 200 μL-sample of the yeast culture followed by centrifugation at 15,000 rpm for 5 min to remove any cells. A total of 100 μL of the supernatant was mixed with 100 μL of MQ water containing 10 mM acetone as internal standard in a 10-mL gas tight vial. The final solution was analyzed on a Shimadzu GC 2010 gas chromatograph equipped with an HS-20 autosampler (Shimadzu). The sample vials were heated at 60°C for 6 min to allow evaporation of the esters and alcohols to the headspace of the vial. A total of 1 mL of the headspace was then injected on a DB-WAX UI column (30 m length, 0.53 mm inner diameter, 1 μm film thickness, Agilent) with a split ratio 1:20. The column temperature was kept at 50°C for 1 min followed by an increase to 90°C at a rate of 5°C min^–1^ followed by an increase to 230°C at a rate of 230°C min^–1^ where the temperature was kept at 230°C for 1 min.

Cultures of transformed *C. beijerinckii* (pCOSCB3:EV or pCOSCB3:WanEat1) were analyzed for *in vivo* alcoholysis by sampling 200 μl of the anaerobic culture followed by centrifugation at 15,000 rpm for 5 min. A total of 100 μL of the supernatant was mixed with 100 μL of MQ water containing 10 mM acetonitrile as internal standard in a 10-mL gas tight vial. Samples were analyzed as described above. The column temperature was kept at 70°C for 1 min followed by an increase to 125°C at a rate of 50°C min^–1^ followed by an increase to 230°C at a rate of 70°C min^–1^ where the temperature was kept at 230°C for 1 min.

All the data presented in this study are averages of biological triplicates and the standard deviation is presented as the error bar in all figures.

### Eat1 Homolog Accession Numbers

The accession numbers of the Eat1 homologs used in this study are: *W. anomalus* Eat1 (XP_019041020.1), *W. ciferii* Eat1 (XP_011273049.1), *K. marxianus* Eat1 (KMAR_10772), *K. lactis* Eat1 (KLLA0_E24421g), *C. jadinii* Eat1 (CEP25158.1), *C. fabianii* Eat1(CDR40574.1), *H. uvarum* Eat1 (D499_0A01740), and *S. cerevisiae* Eat1 (YGR015C).

## Results

### Eat1 Accelerates 4-Nitrophenol Release Through Alcoholysis

**FIGURE 3:**
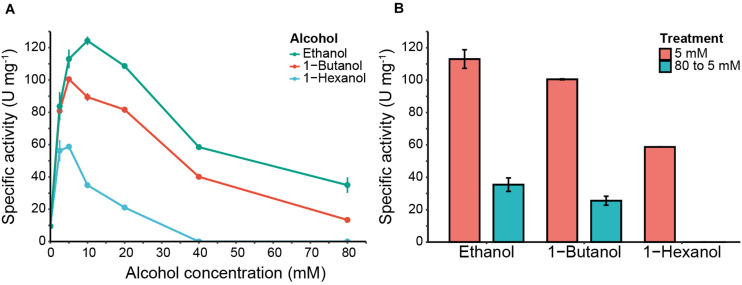
4-nitrophenol release and Eat1 inhibition assays in the presence of different alcohols. **(A)** 4-nitrophenol release by Eat1 was measured colorimetrically using 100 μM 4-nitrophenyl acetate and different concentrations (0–80 mM) of ethanol (blue line), 1-butanol (red line) or 1-hexanol (green line). **(B)** Inhibition of Eat1 was assessed by exposing Eat1 to 80 mM of ethanol, 1-butanol or 1-hexanol and immediately diluting it to 5 mM final concentration (blue bars). The release of 4-nitrophenol was monitored and compared with an assay where pre-treatment of Eat1 with 80 mM alcohols was omitted (red bars). Error bars indicate the standard deviation. 1 U = 1 μmol min^– 1^.

The recently discovered α/β-hydrolase Eat1 exhibits three activities: AAT, esterase and thioesterase (Kruis et al., 2017). Kruis et al. (2017) showed that the esterase and thioesterase activities are decreased by high concentrations of ethanol. This could be due to either inactivation of the enzyme because of unfolding or preference of the enzyme for alcohols over water as acyl acceptors. Together with the ability of the enzyme to use esters and thioesters as substrates, we hypothesized that Eat1 should also be capable of catalyzing alcoholysis (and thiolysis). To demonstrate the predicted alcoholysis, we set-up a classic esterase assay using 4-nitrophenyl acetate and monitored the release of 4-nitrophenol by Eat1 in the absence or presence of various concentrations (0–80 mM) of ethanol, 1-butanol or 1-hexanol (Figure 3A). This method enabled us also to investigate the effect of high alcohol concentrations on the Eat1 activities.

In the absence of alcohols, Eat1 catalyzed the release of 4-nitrophenol with a specific activity of 9.52 ± 0.49 U mg^–1^, representing its esterase activity. Interestingly, the release of 4-nitrophenol increased dramatically when ethanol, 1-butanol or 1-hexanol were added to the reaction mixture. A maximum specific activity was reached with 10 mM ethanol (124.30 ± 2.54 U mg^–1^), 5 mM 1-butanol (100.49 ± 0.27 U mg^–1^) or 2.5 mM 1-hexanol (60.25 ± 0.88 U mg^–1^).

The observed acceleration of the 4-nitrophenol release confirms our assumption that Eat1 has a preference for alcohols as acyl acceptors. As such, Eat1 performs alcoholysis instead of hydrolysis in the presence of alcohols. To confirm alcoholysis over hydrolysis and to exclude non-specific acceleration of 4-nitrophenol release, we incubated 2.5 mM of 4-nitrophenyl acetate with 2.5 mM of ethanol in the presence of 0.0138 mg mL^–1^ Eat1 and analyzed the products by GC. As expected, ethyl acetate was the major product when 4-nitrophenyl acetate and ethanol were used as substrates (Supplementary Figure S1). Also, alcoholysis dominated over hydrolysis as ethyl acetate reached the maximum conversion of 2.5 mM. Similarly, butyl acetate and hexyl acetate were detected by GC (data not shown). These esters were not detected in the control samples that did not contain alcohols or where the Eat1 enzyme was omitted from the assay.

### High Alcohol Concentration Irreversibly Inhibits Eat1

Whereas a low alcohol concentration increased the release of 4-nitrophenol, higher alcohol concentrations (>10 mM) caused a decrease in the specific activity of Eat1 for all three tested alcohols (Figure 3A). This reduction was more apparent at a concentration of 80 mM for ethanol and 1-butanol where the specific activity for 4-nitrophenol release was 23.95 ± 4.81 and 13.41 ± 0.84 U mg^–1^, respectively. For 1-hexanol, the release of 4-nitrophenol was abolished at 40 mM or higher.

The observed inhibition of 4-nitrophenol release with increased alcohol concentrations suggested that the alcohols irreversibly inhibit Eat1. To assess this, we repeated our previous experiment for 4-nitrophenol release, but we first incubated Eat1 with 80 mM of ethanol, 1-butanol or 1-hexanol and immediately diluted it to a final alcohol concentration of 5 mM, before starting the assay. All other constituents of the assay mixture were the same as in the standard assay. Our results demonstrate that the Eat1 enzyme was irreversibly inhibited by alcohols as it was not able to recover its activity after exposure to high alcohol concentrations (Figure 3B).

### Eat1 Catalyzes Alcoholysis With Various Alcohols in an Aqueous Environment

Our previous results confirmed alcoholysis by Eat1 in the presence of 4-nitrophenyl acetate and alcohols in an aqueous environment. As Eat1 is present in various ester-producing yeasts, it is important to determine the alcohol (and acyl) specificity of Eat1. Whereas 4-nitrophenyl acetate is an excellent compound for quantifying alcoholysis, it is not a physiologically relevant compound. For this reason, we replaced 4-nitrophenyl acetate with ethyl acetate (the main ester found in yeasts having Eat1) as the acyl donor in our assays and determined the alcohol specificity of Eat1 using various primary, secondary or tertiary alcohols (C1–10) as acyl acceptors (Table 3).

**TABLE 3:** Specific activity of Eat1 toward various primary, secondary and tertiary alcohols during alcoholysis with ethyl acetate as the acyl donor.

Alcohol carbon length	Alcohol name	XLogP3	Specific activity (U mg^–1^)
**Primary**			
3	1-Propanol	0.30	7.56 ± 1.27
4	1-Butanol	0.90	4.34 ± 1.76
5	1-Pentanol	1.60	1.51 ± 0.72
6	1-Hexanol	2.00	1.20 ± 0.55
6	Cis-3-Hexenol	1.30	2.14 ± 0.32
6	Trans-2-Hexenol	1.40	1.81 ± 0.70
8	2-Phenylethanol	1.40	2.19 ± 1.04
8	1-Octanol	3.00	0.35 ± 0.08
9	1-Nonanol	4.30	0.10 ± 0.02
10	1-Decanol	4.60	0.18 ± 0.02
10	Citronellol	3.20	0.36 ± 0.02
10	Geraniol	2.90	0.43 ± 0.01
**Secondary**			
3	2-Propanol	0.30	0.75 ± 0.31
5	2-Pentanol	1.20	0.26 ± 0.07
8	1-Phenylethanol	1.40	0.45 ± 0.07
8	1-Octen-3-ol	2.60	ND
9	3-Methyloctan-4-ol	3.10	ND
10	Carveol	2.10	ND
**Tertiary**			
5	2-Methyl-3-butyn-2-ol	0.30	ND
9	2-phenyl-2-propanol	1.80	ND
10	Linalool	2.70	ND

*ND, not detected; 1 U = 1 μmol min^–1^; ± indicates the standard deviation; the XLogP3 value was derived from https://pubchem.ncbi.nlm.nih.gov/.*

Eat1 was rather promiscuous with respect to the alcohols. Alcoholysis was observed using various primary and secondary alcohols. 1-Propanol was the best short chain primary alcohol acceptor with a specific activity of 7.56 ± 1.27 U mg^–1^ whereas longer chain primary alcohols, such as 1-nonanol and 1-decanol, showed a specific activity of only 0.10 ± 0.02 and 0.18 ± 0.02 U mg^–1^, respectively. More complex primary alcohols, such as 2-phenylethanol (2.19 ± 1.04 U mg^–1^), citronellol (0.36 ± 0.02 U mg^–1^) and geraniol (0.43 ± 0.01 U mg^–1^), were also accepted by Eat1 even with higher rate than some simple primary alcohols.

The short chain secondary alcohols 2-propanol (0.75 ± 0.31 U mg^–1^), 2-pentanol (0.26 ± 0.07 U mg^–1^), and 1-phenylethanol (0.45 ± 0.07 U mg^–1^) were accepted during alcoholysis by Eat1 but longer complex secondary alcohols such as 1-octen-3-ol, 3-methyloctan-4-ol, and carveol did not show any activity with Eat1. No activity was detected with any of the tertiary alcohols tested.

After determining the substrate specificity of Eat1 toward alcohols using ethyl acetate as the acyl donor, we further investigated the acyl specificity of the alcoholysis reaction (Table 4). Ethyl acetate, ethyl butyrate, ethyl valerate, or ethyl hexanoate were used as the acyl donors and 1-butanol was used as the acyl acceptor. Ethyl acetate was the preferred acyl donor for Eat1 as a higher specific activity (4.34 ± 1.76 U mg^–1^) was observed compared to ethyl butyrate (1.70 ± 0.43 U mg^–1^). No alcoholysis activity was found using ethyl valerate and ethyl hexanoate.

**TABLE 4:** Acyl specificity of Eat1 during alcoholysis.

Acyl carbon length	Ethyl ester donor	Butyl ester product	Specific activity (U mg^–1^)
2	Ethyl acetate	Butyl acetate	4.34 ± 1.76
4	Ethyl butyrate	Butyl butyrate	1.70 ± 0.43
5	Ethyl valerate	Butyl valerate	ND
6	Ethyl hexanoate	Butyl hexanoate	ND

*ND, not detected. 1 U = 1 μmol min^–1^; ± indicates the standard deviation.*

### *In vivo* Alcoholysis in WT and Engineered Yeasts

Alcoholysis was previously demonstrated in cell free extract and *in vivo* in certain lactic acid bacteria (Liu et al., 2003, 2004; Mukdsi et al., 2009; Mukdsi et al., 2018). Since Eat1 homologs are present in various yeasts, we assessed the capacity of such yeasts to perform *in vivo* alcoholysis. We selected *S. cerevisiae* (control) and several Crabtree negative yeasts and grew them in YMM-no-iron in the presence of 5 mM butyl butyrate. *S. cerevisiae* was selected as a control because it is Crabtree positive and therefore does not follow the hypothesis of acetyl-CoA accumulation into the mitochondria and overexpression of Eat1 during iron starvation. Our selection of Crabtree negative yeasts and the indicated growth conditions are based on the following: i) the selected yeasts have active homologs of Eat1, ii) Crabtree negative yeasts show enhanced ethyl acetate production during iron limitation which is hypothesized to be correlated with acetyl-CoA accumulation and Eat1 overexpression in the mitochondria, iii) butyl butyrate, butanol and butyrate are not produced by the selected yeasts and thus alcoholysis between the supplemented butyl butyrate and the endogenously produced ethanol can be easily screened through the production of ethyl butyrate (Urit et al., 2012; Kruis et al., 2017, 2018). As a control for potential esterification between free butyric acid and ethanol, we replaced butyl butyrate in the growth medium with 5 mM butyrate.

**FIGURE 4:**
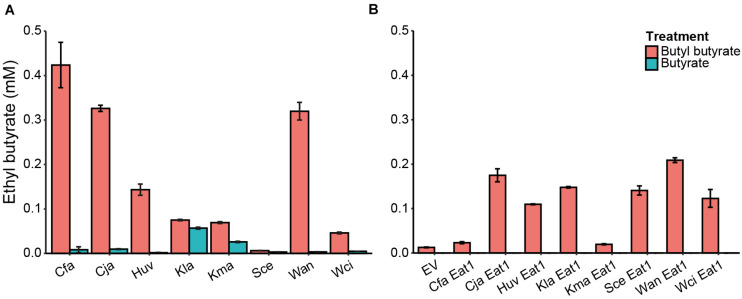
*In vivo* alcoholysis in wild type yeasts or in engineered *S. cerevisiae*. **(A)** *In vivo* alcoholysis by WT yeasts grown in the presence of 5 mM butyl butyrate (red bars) or 5 mM butyrate (blue bars) for 48 h. *n* = 3. **(B)** Screening of different Eat1 homologs for their capacity for alcoholysis when expressed in *S. cerevisiae* grown for 24 h in the presence of 5 mM butyl butyrate (red bars) or 5 mM butyrate (blue bars). *n* = 3. EV, empty vector; Cfa, *Cyberlindnera fabianii*; Cja, *Cyberlindnera jadinii*; Huv, *Hanseniaspora uvarum*; Kla, *Kluyveromyces lactis*; Kma, *Kluyveromyces marxianus*; Sce, *Saccharomyces cerevisiae*; Wan, *Wickerhamomyces anomalus*; Wci, *Wickerhamomyces ciferrii*. The error bars indicate standard deviations.

Ethyl butyrate was produced by all tested yeasts in cultures containing butyl butyrate (Figure 4A). *C. fabianii*, *C. jadinii* and *W. anomalus* showed the highest production of ethyl butyrate (0.42 ± 0.05, 0.33 ± 0.01 and 0.32 ± 0.02 mM, respectively) whereas *K. lactis*, *K. marxianus*, and *W. ciferii* produced very little ethyl butyrate (<0.1 mM). *H. uvarum* was an intermediate ethyl butyrate producer (0.14 ± 0.01 mM) whereas the *S. cerevisiae* control produced only traces of ethyl butyrate. Yeast cultures containing free butyrate, instead of butyl butyrate, showed low ethyl butyrate production (<0.06 mM).

Our results indicate that yeast species are capable of *in vivo* alcoholysis, but that the capacity varies among yeast species. This might be correlated to the expression level of Eat1 under the defined conditions or the ability of the different Eat1 homologs (or other enzymes) to perform alcoholysis in the first place. To examine whether different Eat1 homologs have a different alcoholysis capacity *in vivo*, we developed a more robust assay where we overexpressed these homologs in *S. cerevisiae* under the inducible CUP1 promoter. These recombinant *S. cerevisiae* strains were grown in the presence of 5 mM butyl butyrate to assess alcoholysis or in the presence of 5 mM butyrate to assess esterification.

Ethyl butyrate was produced in all *S. cerevisiae* variants (Figure 4B). However, Cja-Eat1, Huv-Eat1, Kla-Eat1, Sce-Eat1, Wan-Eat1, and Wci-Eat1 produced higher ethyl butyrate levels compared to the other homologs and the empty vector control. Wan-Eat1 was the best ethyl butyrate producing enzyme reaching 0.21 ± 0.01 mM. Surprisingly, Cfa-Eat1 did not show high ethyl butyrate production through alcoholysis despite the observed alcoholysis in the WT yeast assays. Furthermore, Kla-Eat1, Sce-Eat1, and Wci-Eat1 could perform *in vivo* alcoholysis when overexpressed in *S. cerevisiae* even though the WT yeasts did not show this capacity. Control cultures containing butyrate did not produce ethyl butyrate through esterification.

### Ester Upgrade Through *in vivo* Alcoholysis in *Clostridium beijerinckii*

The ability of Eat1 to perform *in vivo* alcoholysis may provide an opportunity to upgrade low value esters with high value alcohol moieties *in vivo* and therefore increase their commercial value. To realize this, we chose *C. beijerinckii* NCIMB 8052 as the appropriate host for ester upgrading since it is a natural producer of butanol and thus a good candidate to produce butyl esters through alcoholysis.

**FIGURE 5:**
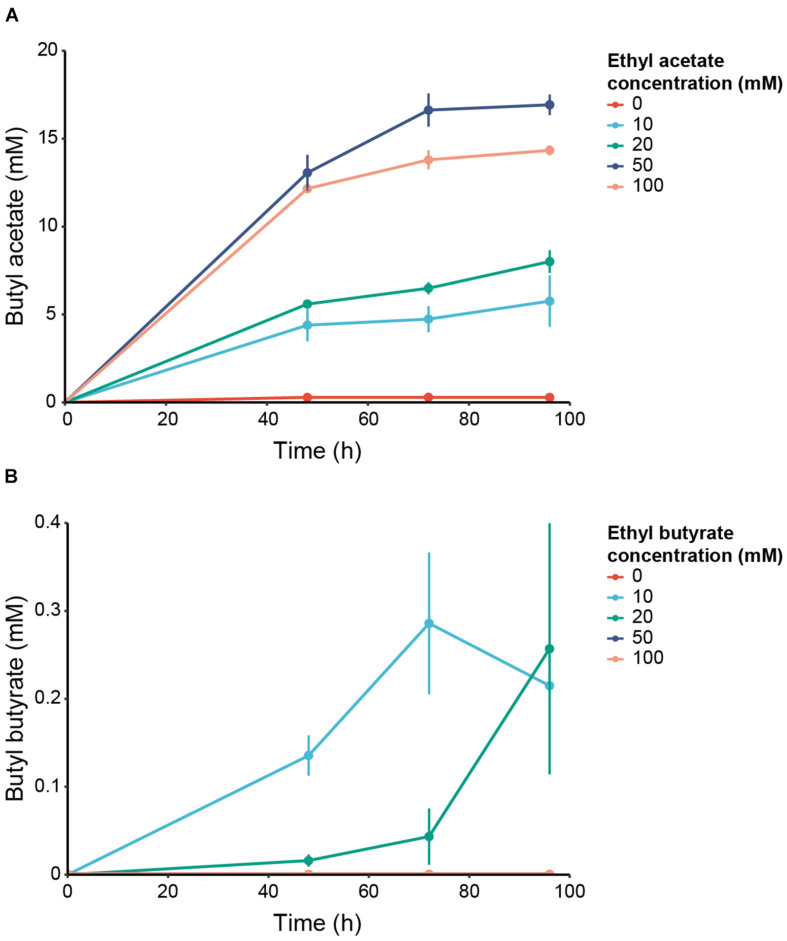
*In vivo* alcoholysis in engineered *C. beijerinckii*. **(A)** Production of butyl acetate by *C. beijerinckii* expressing WT WanEat1 and grown in 0 (red), 10 (light blue), 20 (green), 50 (dark blue) or 100 mM (orange) ethyl acetate. **(B)** Production of butyl butyrate by *C. beijerinckii* expressing WT WanEat1 and grown in 0 (red), 10 (light blue), 20 (green), 50 (dark blue), or 100 mM (orange) ethyl butyrate. Error bars indicate the standard deviation.

Batch cultures of *C. beijerinckii* transformed either with an empty plasmid (pCOSCB:EV) or with a plasmid constitutively expressing WanEat1 (pCOSCB3:WanEat1) were grown in the presence of various concentration of ethyl acetate (0–100 mM). Without the supplementation of ethyl acetate, the Eat1 expressing cultures produced only traces of butyl acetate (Figure 5A). However, high levels of butyl acetate was produced by all cultures containing ethyl acetate and expressing Eat1. The highest production of butyl acetate (16.92 ± 0.58 mM) was reached when 50 mM ethyl acetate was supplemented in the medium after 96 h of fermentation. A total of 100 mM ethyl acetate seemed to have a toxic effect on *C. beijerinckii* as butyl acetate production decreased. Empty vector control cultures did not show butyl acetate production when ethyl acetate was supplied in the growth medium (data not shown).

In addition to acetate esters, Eat1 can also catalyze alcoholysis with butyrate esters. To assess whether we can upgrade even more complex ethyl esters to butyl esters, we performed a similar experiment as reported above, but supplied ethyl butyrate as the acyl donor at different concentrations (0–100 mM). Butyl butyrate was produced in cultures expressing Eat1 (Figure 5B). Maximum production of 0.29 ± 0.08 mM was reached in cultures supplemented with 10 mM ethyl butyrate and grown for 72 h. *C. beijerinckii* could not grow in cultures containing ethyl butyrate concentrations equal to or higher than 50 mM. Obviously, butyl butyrate was not detected in those cultures, nor in cultures bearing the empty plasmid.

### Eat1 Can also Perform Thiolysis

Eat1 can accept esters and thioesters as acyl donors. This triggered us to investigate the capacity of Eat1 to accept thiols as acyl acceptors and therefore perform thiolysis. To assess this, we used the 4-nitrophenol release assay developed for alcohols, but instead we replaced the alcohols with either ethanethiol or butanethiol.

**FIGURE 6:**
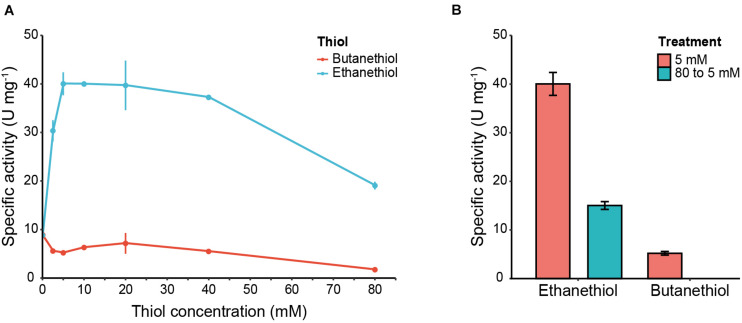
4-nitrophenol release assay and Eat1 inhibition in the presence of thiols. **(A)** Effect of 4-nitrophenol release from 4-nitrophenyl acetate incubated with various concentrations (0–80 mM) of ethanethiol or butanethiol. **(B)** Inhibition assay of Eat1 by exposing Eat1 to 80 mM ethanethiol or butanethiol followed by immediate dilution to a final concentration of 5 mM. Error bars indicate the standard deviation. 1 U = 1 μmol min^– 1^.

The release of 4-nitrophenol from 4-nitophenyl acetate was accelerated in the presence of ethanethiol (Figure 6A). The production of ethyl thioacetate was confirmed by GC (data not shown). This clearly indicates that Eat1 is also capable of catalyzing thiolysis (Figure 6A). The release of 4-nitrophenol in the presence of increasing concentrations of ethanethiol followed a similar trend as observed for alcoholysis (Figure 3A); 4-nitrophenol release is enhanced (40.04 ± 2.36 U mg^–1^) at low ethanethiol concentrations (5 mM) but it is decreased (19.06 ± 0.80 U mg^–1^) when a high (80 mM) concentration is used. Using a similar setup as used in the alcoholysis assays, we could show that the lower thiolysis activity at higher ethanethiol concentrations was a result of the irreversible inhibition of Eat1 by ethanethiol (Figure 6B). In contrast to ethanethiol, butanethiol did not accelerate 4-nitrophenol release when it was added to the reaction medium. On the other hand, it slightly decreased the activity of Eat1 toward 4-nitrophenyl acetate compared to its esterase activity.

For completeness, we performed *in vitro* thiolysis between ethyl acetate as the acyl donor and ethanethiol as the acyl acceptor (Table 5). Also, for comparison, we performed alcoholysis between ethyl thioacetate as the acyl donor and ethanol as the acyl acceptor. Ethyl thioacetate was produced through thiolysis and ethyl acetate was produced through alcoholysis. However, the specific activity to produce these compounds differed based on the acyl donor. When ethyl acetate was the acyl donor, a very low specific activity was observed with ethanethiol (0.08 ± 0.01 U mg^–1^) as the acyl acceptor. In contrast, the specific activity of Eat1 increased when ethyl thioacetate was used as the acyl donor and ethanol as the acceptor (6.27 ± 0.86 U mg^–1^).

**TABLE 5:** Comparison between thiolysis and alcoholysis.

Acyl donor	Acyl acceptor	Product	Specific activity (U mg^–1^)
Ethyl acetate	Ethanethiol	Ethyl thioacetate	0.08 ± 0.01
Ethyl thioacetate	Ethanol	Ethyl acetate	6.27 ± 0.86

*1 U = 1 μmol min^–1^; ± indicates the standard deviation.*

### Comparison of Eat1 Catalytic Activities

Previously, the Eat1 enzyme was reported to exhibit three types of catalytic activities *viz*. AAT, esterase, and thioesterase activity (Kruis et al., 2017). In Table 6, we compare the novel alcoholysis and thiolysis activities to the previously reported Eat1 activities. The specific alcoholysis activity is ∼450-fold higher than the AAT activity when ethyl acetate or acetyl-CoA was used as the acyl donor, respectively. Alcoholysis is also the highest activity when 4-nitrophenyl acetate was used as the acyl donor, showing the clear preference of Eat1 to alcohols over thiols and water (alcohol > thiol > water). The thiolysis activity between ethyl acetate and ethanethiol, although ∼5-fold higher than the AAT activity, is ∼95-fold lower than the alcoholysis activity between ethyl acetate and 1-propanol.

**TABLE 6:** Comparison between the different activities of Eat1.

Activity	Acyl donor	Acyl acceptor	Specific activity (U mg^–1^); (fold difference compared to AAT)	Source
AAT	Acetyl-CoA	Ethanol	0.017; (1)	Kruis et al. (2017)
Thioesterase	Acetyl-CoA	H_2_O	0.032; (1.88)	Kruis et al. (2017)
Esterase	Ethyl acetate	H_2_O	0.85; (50)	Kruis et al. (2017)
Esterase	4-nitrophenyl acetate	H_2_O	10.7; (629)	This study
Alcoholysis	4-nitrophenyl acetate	Ethanol	112; (6588)	This study
Alcoholysis	Ethyl acetate	1-Propanol	7.56; (445)	This study
Thiolysis	4-nitrophenyl acetate	Ethanethiol	40.04; (2355)	This study
Thiolysis	Ethyl acetate	Ethanethiol	0.08; (4.7)	This study

*1 U = 1 μmol min^–1^.*

## Discussion

In this study we show that the α/β-hydrolase Eat1 can catalyze both alcoholysis and thiolysis reactions. This discovery extends the catalytic portfolio of Eat1 to five different activities: AAT, esterase, thioesterase, alcoholysis and thiolysis (Figure 1). Although all five activities most likely use the same canonical esterase mechanism, the acyl donor and the acyl acceptor affinity and specificity defines which of the activities prevails. For example, *in vitro*, AAT is dominant over the esterase and the thioesterase activities as Eat1 favors ethanol over water in an aqueous environment (Kruis et al., 2017). The preference of Eat1 for alcohols and thiols also explains that alcoholysis and thiolysis prevail over hydrolysis in an aqueous environment. This was apparent when we tested the release of 4-nitrophenol from 4-nitrophenyl acetate by using Eat1 in an aqueous environment. In the absence of alcohols or thiols, the rate of hydrolysis is the only determinant for the half-life of the acyl-enzyme intermediate (Müller et al., 2020). However, when alcohols or thiols were present, Eat1 preferred the alcohol or thiol nucleophile over water. This resulted in a shorter half-life of the acyl-enzyme intermediate that in turn resulted in an overall faster release of 4-nitrophenol.

In the presence of high alcohol or thiol concentrations (>10 mM), Eat1 reduced the release of 4-nitrophenol from 4-nitrophenyl acetate compared to lower concentrations (<10 mM). A similar trend was also observed by other studies when different acyltransferases were tested for their capacity to catalyze alcoholysis or transesterification (Chulalaksananukul et al., 1992; Rizzi et al., 1992; El Rassy et al., 2004; Müller et al., 2020). The reduced activity of Eat1 by high alcohol or thiol concentrations suggested that substrate inhibition occurs. Our results, clearly demonstrate that Eat1 was irreversibly inhibited *in vitro* when exposed to high concentrations of either alcohols or thiols. The irreversible ping-pong bi-bi mechanism may explain our observations (Cheng and Tsai, 2003; Yadav and Lathi, 2003; Varma and Madras, 2008). According to this mechanism, the alcohol acts as an inhibitor of the enzyme by binding to the free enzyme thus forming ineffective, dead-end complexes. Our results obey this mechanism as Eat1 cannot recover its activity even after diluting the alcohol or thiol concentration. However, unfolding of Eat1 at high alcohol concentration should not be excluded as a possible explanation for our observations. Nevertheless, inhibition of Eat1 at 80 mM of ethanol is surprising as the ethanol production by the yeasts *K. marxianus* and *K. lactis* may exceed 100 mM (Kruis et al., 2018). Still, Eat1 has been shown to be the main enzyme that contributes to high ethyl acetate production in both yeasts (Kruis et al., 2018; Löbs et al., 2018). Compartmentalization of Eat1 to the yeast mitochondria and low ethanol concentration inside the mitochondria may assist in preventing the inhibition of Eat1 by ethanol *in vivo*.

The novel alcoholysis activity of Eat1 described here, exceeds the former activities (AAT, esterase and thioesterase) by far. The specific alcoholysis activity is ∼450-fold higher than the AAT activity and ∼9-fold higher than the esterase activity (Table 6). Although these activities depend on the assay conditions, the enormous differences between the activities detected *in vitro* strongly suggest that alcoholysis does play a role *in vivo* as well. Because of the high alcoholysis activity, ethyl acetate may even outcompete acetyl-CoA as acyl donor, resulting in the recycling of ethyl acetate instead of the *de novo* synthesis from acetyl-CoA. In other words, ethyl acetate may occupy the active site and as such it would prevent access of acetyl-CoA. To avoid such a futile cycle of ethyl acetate synthesis, gas stripping can be applied to remove ethyl acetate from the medium as much as possible and allow access of acetyl-CoA to the active site of Eat1. The effect of alcoholysis may have played a role in a recent study in which Eat1 was used as an AAT to produce ethyl acetate in *E. coli* (Bohnenkamp et al., 2020). In this previous study, it was reasoned that gas stripping would enhance ethyl acetate production by preventing its hydrolysis by Eat1. However, the alcoholysis activity of Eat1 was not known at that time. Hence, in retrospect, the insights gained in the present study indicate that gas stripping not only resulted in enhanced ethyl acetate production by avoiding hydrolysis, but most importantly, by avoiding alcoholysis. In total 42.8 mM of ethyl acetate was produced, which is the highest ethyl acetate production in an engineered microorganism to date (Bohnenkamp et al., 2020).

Eat1 is able to accept a broad range (C3 to C10) of primary and secondary alcohols, although with different specific activities. One explanation of the observed differences may be the variable solubility of each alcohol in water since their XLogP3 values follow a comparable pattern as the specific activity by Eat1 during alcoholysis (Supplementary Figure S2). Nevertheless, this may explain only the trends observed for primary alcohols, as such a correlation did not apply to secondary alcohols. Therefore, especially for the secondary alcohols, the difference in specific activity is likely due to steric hindrance of the alcohol moieties in the Eat1 substrate-binding pocket (Hoydonckx et al., 2004; Cha et al., 2019). The interplay between the catalytic pocket and the substrates of Eat1 can only be resolved with the crystal structure of Eat1. Unfortunately, we have not yet succeeded in obtaining suitable Eat1 crystals.

With respect to acyl specificity, Eat1 can accept only short acyl chains (C2 and C4) and shows no activity toward longer acyl chains (C5 and C6). The wide alcohol and the narrow acyl specificity of Eat1, differentiates it from the yeast Eht1 and Eeb1 α/β-hydrolases. Eht1 and Eeb1 also exhibit AAT activity but their acyl specificity is broad (C4 to C12). Similar to Eat1, Eht1 and Eeb1 also have hydrolytic activities (Saerens et al., 2006). Since Eht1 and Eeb1 can hydrolyze and produce esters following the same canonical esterase mechanism as Eat1, it can also be expected that Eht1 and Eeb1 can perform alcoholysis. The extent to which activity prevails depends on the substrate specificity and availability and on the hydrophobicity of the catalytic pocket of the enzyme (Mathews et al., 2007; de Leeuw et al., 2018). Future experiments should include Eht1 and Eeb1 as potential enzymes for alcoholysis.

It was not until recently that the Eat1 enzyme was included in the list of AATs responsible for the production of esters in yeasts, including *S. cerevisiae*, *W. anomalus*, *K. marxianus*, *K. lactis*, and others. Although Eat1 was demonstrated as an important AAT *in vivo*, its role for *in vivo* alcoholysis was not known. Alcoholysis, if present, can play an important role in the production and distribution of esters in yeasts. Such a role has previously been described for lactic acid bacteria where alcoholysis is the main mechanism for the production of esters (Liu et al., 2003; Mukdsi et al., 2009; Mukdsi et al., 2018). Similarly, we hypothesize that yeasts expressing Eat1 homologs may perform alcoholysis *in vivo*. As shown here, *C. fabianii*, *C. jadinii*, and *W. anomalus* could indeed perform *in vivo* alcoholysis to recombine supplemented butyl butyrate and endogenously produced ethanol, under iron-limited conditions. Surprisingly, *K. lactis* and *K. marxianus* did not show *in vivo* alcoholysis even though their Eat1 homolog is the main contributor for the production of ethyl acetate (Kruis et al., 2017; Löbs et al., 2018). Although Eat1 was highly expressed in *W. anomalus* under iron-limited conditions, we do not have transcriptome data to support high expression of Eat1 for all the yeasts tested in this study (Kruis et al., 2017). Therefore, it is unclear whether the Eat1 homologs are better expressed under iron-limited conditions in all yeasts even though ethyl acetate production is boosted in iron-limited conditions (Kruis et al., 2018). In addition, the specificity of each Eat1 homolog toward different acids and alcohols is not known, and hence, the availability, accessibility and processing of esters and alcohols may differ between yeast species.

When we overexpressed Eat1 homologs in *S. cerevisiae*, several of the transformed strains were also able to catalyze *in vivo* alcoholysis. In the case of *C. jadinii*, *H. uvarum*, and *W. anomalus* Eat1, our results agree with the observed alcoholysis in the WT yeasts, which may suggest that the Eat1 homolog is the main enzyme for alcoholysis. However, at present, no firm conclusion can be drawn since Eat1 knock-out strains could not be obtained. While WT *K. lactis*, *S. cerevisiae*, and *W. ciferii* did not show alcoholysis, overexpression of their respective Eat1 homolog in *S. cerevisiae* did result in alcoholysis. This observation further strengthens the hypothesis that other factors affect the alcoholysis capacity of these yeasts such as the expression levels of Eat1 during the defined growth conditions, the availability of the substrate and the metabolic features of the different yeast species. Altogether, these *in vivo* experiments show that various Eat1 homologs, other than the Eat1 tested in this study (*W. anomalus*), are capable of alcoholysis, but that the expression level and the specificity determines the final alcoholysis capacity of the corresponding strains. Our results further show that ester production in yeasts is not simply the result of AAT activity of Eat1, Eht1, Eeb1 and Atf1, but also of the alcoholysis activity of Eat1. This observation may revolutionize the food and beverage industry as the production of esters through alcoholysis has been completely neglected. The supplementation of short chain triglycerides (e.g., tributyrin) in the growth medium may provide the acyl substrate for the Eat1 enzyme and the subsequent production of novel esters by yeasts. We foresee that this discovery will enable food and beverage producers to further innovate with their yeast strains and their substrates in their fermentation processes and to produce novel food and beverage products.

As an alternative to current unsustainable processes, various AATs combined with extensive metabolic engineering have been used to produce esters in microorganisms (Kruis et al., 2019). In our study, we applied a different approach for *in vivo* ester production by taking advantage of the alcoholysis activity of Eat1. We showed that engineered *C. beijerinckii* strains overexpressing Eat1 could upgrade ethyl acetate or ethyl butyrate to butyl acetate or butyl butyrate, respectively, through alcoholysis. The observed difference between the production level of butyl acetate and butyl butyrate is probably due to the acyl specificity of Eat1, as it has a higher specific activity with acetate esters than with butyrate esters (Table 2). In addition, we hereby show that the availability of the acyl donor (i.e., acyl-CoA) in *C. beijerinckii* is the main factor that controls the production of esters. Strains without the supplementation of ethyl esters produced only traces of butyl esters through AAT, indicating that the acyl-CoA availability is limiting for the AAT reaction. This observation was also made in a recent study where the availability of acyl-CoAs in *C. saccharoperbutylacetonicum* was the limiting factor for ester production (Feng et al., 2020). Our data show that *in vivo* ester upgrade through alcoholysis provides an alternative to traditional ester production methods by avoiding the limitation of the acyl-CoA availability. Such an approach may open new research lines for the production of esters using microorganisms.

Finally, we discovered that Eat1 also shows thiolysis activity. Thiolysis is known to be catalyzed by lipases from *R. miehei* (Lipozyme IM 20^®^), *C. antarctica* (Novozym 435^®^), or *Thermomyces lanuginosus* for the production of thiobutyl butyrate, thiobutyl valerate, thiostearyl palmitate, thiohexyl octanoate and other acetate thioesters (Zaks and Klibanov, 1985; Cavaille-Lefebvre and Combes, 1997; Weber et al., 1999; Hedfors et al., 2010; Zhou et al., 2018). To our knowledge, this is the first time that an esterase was shown to catalyze thiolysis of short chain thioesters. As thiols, like cysteine and glutathione, are present in yeasts, this activity may also be of physiological relevance or play a role in the production of thiol-based aromas. Further research is needed to assess the importance of thiolysis by Eat1 and its homologs for the *in vitro* and *in vivo* production of thioesters.

## Data Availability Statement

The raw data supporting the conclusions of this article will be made available by the authors, without undue reservation.

## Author Contributions

CP, RW, and SK conceived and designed the experiments. CP, LL, and IC performed the experiments. MF and JVdO gave advice for the experimental design. CP, RW, and SK wrote the manuscript. All the authors have reviewed and approved the present manuscript. All the authors have contributed significantly to realize this work.

## Conflict of Interest

The authors declare that the research was conducted in the absence of any commercial or financial relationships that could be construed as a potential conflict of interest.
