# Supplementary material for: Eat1-Like Alcohol Acyl Transferases From Yeasts Have High Alcoholysis and Thiolysis Activity
Source: Front Microbiol. 2020 Oct 29;11:579844. doi: 10.3389/fmicb.2020.579844 (PMC7658179; doi:10.3389/fmicb.2020.579844)
Supplement: Supplementary file 1 [file Data_Sheet_1.docx]

**Supplementary Figure 1.** Detection of ethyl acetate in the presence of 2.5 mM 4-nitrophenyl acetate as acyl donor and 2.5 mM ethanol as acyl acceptor. 0.0318 mg ml^-1^ Eat1 was used.

**Supplementary Figure 2.** Specific activity of Eat1 with different alcohols against their XLogP3 value. The XLogP3 value was derived from <https://pubchem.ncbi.nlm.nih.gov/>.
